# Molecular diagnosis and phylogenetic analysis of a Middle East respiratory syndrome coronavirus human case in Jordan

**DOI:** 10.1093/eurpub/ckae106

**Published:** 2025-01-13

**Authors:** Ehab A Abu-Basha, Zuhair B Ismail, Mohammad H Alboom, Ayesha Alkofahi, Basil H Amarneh, Omar Al-Omari, Alaa Fahmawi, Abdullah Alshammari, Mais Lakaideh, Shereen Shaban, Bilal Al-Omari, Hani Talafha, Zaidoun Hijazeen, Yasmin Daradkeh, Rabeh El-Shesheny, Ghazi Kayali, Whitney Bagge, William B Karesh

**Affiliations:** Jordan University of Science and Technology, Irbid, Jordan; Jordan University of Science and Technology, Irbid, Jordan; Jordan University of Science and Technology, Irbid, Jordan; Jordan University of Science and Technology, Irbid, Jordan; Jordan University of Science and Technology, Irbid, Jordan; Jordan University of Science and Technology, Irbid, Jordan; Jordan University of Science and Technology, Irbid, Jordan; Jordan University of Science and Technology, Irbid, Jordan; Jordan University of Science and Technology, Irbid, Jordan; Jordan University of Science and Technology, Irbid, Jordan; Jordan University of Science and Technology, Irbid, Jordan; Jordan University of Science and Technology, Irbid, Jordan; Jordan University of Science and Technology, Irbid, Jordan; Jordan University of Science and Technology, Irbid, Jordan; Division of Environmental Research, National Research Centre, Giza, Egypt; Human Link, Dubai, United Arab Emirates; EcoHealth Alliance, New York, NY, United States; EcoHealth Alliance, New York, NY, United States

## Abstract

Middle East respiratory syndrome coronavirus (MERS-CoV) is an important zoonotic pathogen. The aim of this paper is to report one polymerase chain reaction (PCR)-positive case of MERS-CoV in a 27-year-old man who was involved in a nationwide longitudinal surveillance study of certain zoonotic diseases in Jordan including MERS-CoV. Whole-blood and nasal swab samples were collected from the man and five camels in the vicinity of his living area. The samples were subjected to enzyme-linked immunosorbent assay (ELISA) and real-time reverse-transcription PCR (RT-PCR) to detect MERS-CoV-specific antibodies and MERS-CoV genetic material, respectively. Genomic sequencing and phylogenetic analysis were also performed to detect similarities with known strains of the virus in the region. In January 2021, an ongoing surveillance study detected a MERS-CoV-positive nasal swab sample from an asymptomatic male and camels using RT-PCR. Phylogenetically, the MERS-CoV isolated in this case belonged to clade B and is clustered with other strains originating in the Arabian Peninsula. The case report represents the first PCR-positive case of MERS-CoV in an asymptomatic individual in Jordan, indicating active circulation of the virus within the population.

## Introduction

Middle East respiratory syndrome caused by coronavirus (MERS-CoV) has posed significant public health challenges since its emergence in Saudi Arabia in 2012 [1]. This virus, part of the *Betacoronavirus* genus, enters cells by binding its envelope protein (S protein) to the dipeptidyl peptidase 4 (DPP4) receptor, which is abundant in mammal lungs [1]. MERS primarily affects the lower respiratory tract, leading to symptoms such as fever, cough, breathing difficulties, and pneumonia [2]. The disease progresses quickly, often resulting in respiratory failure and acute kidney injury, particularly in older males with underlying conditions [2]. Although prevalent among dromedary camels in the Arabian Peninsula and certain African regions, most human infections have been reported in Saudi Arabia [2]. Locally transmitted, laboratory-confirmed MERS-CoV cases have been reported in around 27 countries worldwide [3–5]. Despite ongoing efforts, effective treatment for MERS-CoV infection remains elusive [6].

In Jordan, approximately 10 000 dromedary camels are raised under nomadic and semi-nomadic conditions. Camels play a vital role in the livelihoods of many communities in Jordan [7]. Camel owners regard these animals as valuable sources of wealth, health benefits, companionship, and commodities, fostering close bonds with them [7].

Recent literature has reported the presence of MERS-CoV genetic material in humans and camel samples in Jordan [8–15]. In 2012, a hospital outbreak of MERS-CoV was identified with a 22% fatality rate, involving six healthcare workers, indicating potential hospital spread of MERS-CoV [14]. Notably, camel contact is a primary risk factor for human MERS transmission [14]. A longitudinal surveillance study in Jordan confirmed widespread MERS-CoV presence in camels, emphasizing the critical need to understand transmission dynamics for effective prevention and control strategies [15].

Molecular characterization of MERS-CoV strains isolated from camels and exposed humans has demonstrated their close relatedness [8, 9]. In this report, MERS-CoV was detected in a nasal swab sample obtained from an asymptomatic man in Jordan. It was necessary to characterize this strain of the virus and compare it to previously isolated and identified strains in Jordan in order to detect circulation trends in human and camel populations, genetic mutations, and potential genetic variations that may influence the transmission dynamics of the virus.

## Methods

### Ethical approvals

The study protocols were reviewed and approved by the Institutional Review Board (IRB) for research on human subjects at the Jordan University of Science and Technology (JUST) in Irbid, Jordan, and by Hummingbird IRB in Needham, Massachusetts, USA, through EcoHealth Alliance. The study protocols involving animals were approved by JUST Institutional Animal Care and Use Committee (IACUC).

## Study area and sampling site

The individual being reported in this paper was part of an ongoing One Health nationwide project (2019–2025) “Reducing the Threat of Middle East Respiratory Syndrome Coronavirus and Avian Influenza in Jordan and Strengthening Regional Disease Surveillance Capacity”. The project is a longitudinal human–animal interface study that aims at understanding the zoonotic transmission risk of MERS-CoV and avian influenza. The study covers five regions across Jordan and includes a study population of 250 exposed and 250 unexposed individuals to camels. Systematic random sampling was employed to collect nasopharyngeal and oropharyngeal swabs and blood samples from humans. Nasal swabs and blood samples are also collected from camels (100) and other livestock including cattle, sheep, and goats, and avian species (100 each). Sampling was performed over 3 rounds each year for a total of 10 rounds during the entire study period.

## Sample collection from human participants

A trained MD and nurse collected approximately 8–10 ml of whole blood and placed it in a plain clotting tube. The sample was then placed on ice packs in a box for transportation to the laboratory within 2 hours. In the laboratory, serum was separated by centrifugation at 10 000 g for 15 minutes and stored in sterile tubes at −80°C for further analysis. A nasopharyngeal sample was collected from individuals, placed in 0.5 ml of TRIzol reagent, and transported to the laboratory on ice and stored at −80°C for further tests.

## Sample collection from camels

Whole-blood samples were obtained from five camels located in close proximity to the individual who tested positive for MERS-CoV. A trained veterinarian collected approximately 8–10 ml of whole blood via jugular venipuncture. Similar to the human samples, camel samples were placed on ice packs in a box and transported to the laboratory within 2 hours. In the laboratory, serum was separated by centrifugation at 10 000 g for 15 minutes and stored in sterile tubes at −80°C for further analysis. A nasopharyngeal sample was also collected from the camels, placed in 0.5 ml of TRIzol reagent, and transported to the laboratory on ice and stored at −80°C for further tests.

### Molecular screening of MERS-CoV

RNA was extracted from nasopharyngeal specimens using Direct-Zol miniprep kit (Zymo Research, USA; Catalog ID R2053) according to the manufacturer’s specifications. Nucleic acid extracts were screened by real-time reverse-transcription polymerase chain reaction (rRT-PCR) for the presence of the MERS-CoV UpE primers targeting the upstream region of the E gene: UpE-Fwd (GCAACGCGCGATTCAGTT), UpE-Rev (GCCTCTACACGGGACCCATA), and UpE-Probe (CTCTTCACATAATCGCCCCGAGCTCG). Samples that tested positive by the UpE rRT-PCR were confirmed by an rRT-PCR targeting the open reading frame b gene, employing the following primers: ORF1b-Fwd (TTCGATGTTGAGGGTGCTCAT), ORF1b-Rev (TCACACCAGTTGAAAATCCTAATTG), and ORF1b-Probe (CCCGTAATGCATGTGGCACCAATGT). Internal control, Beta-Actin, was used with the following primers and probe: Beta-Actin-U-Fwd (CCAACTGGGACGACATGGAGA), Beta-Actin-U-Probe (TGGCACCACACCTTCTACAATGAGCT), and Beta-Actin-U-Rev (GTACATGGCTGGGGTGTTGAA) [2].

### Amplification and nucleotide sequencing of RdRp and spike genes

The complementary DNA (cDNA) was synthesized from extracted RNA using PrimeScript first strand cDNA Synthesis Kit (Takara Bio, France). Then amplification of region of coronavirus partial Spike and RdRp genes was performed using primers ORF1a-Fwd1 (TAGGTTTGTGTGCGTTCCTGAC), ORF1a-Rev1 (GCTGTTTCCATAGCACCAGAGA), ORF1a-Fwd2 (GCCACTAAATTTACTTTGTGGAACTACT), ORF1a-Rev2 (CGAGTAAGCAAAGATGGCATAAGTG), Spike-Fwd (ACCCTGTGTATCCATTGTCCCA), and Spike-Rev (ACGGGAGTATTGAGACATGGTAGA) and using HOT FIREPol^®^ Blend Master Mix Kit (Solis BioDyne OÜ, Estonia). The final PCR amplicons were purified and sequenced with the same primers at the Macrogen sequencing facility (Macrogen, Seoul; South Korea).

### Sequence analysis

The obtained sequences were edited and assembled into consensus contigs using SeqMan DNA Lasergene 7 software (DNASTAR Software, DNASTAR, USA). The sequences of spike and RdRp genes were analysed using the genetic data resources available at the National Center for Biotechnology Information (NCBI). Deduced viral sequences were aligned using the ClustalW algorithm (Thompson et al., 1994). Mega-7 was used for the phylogenetic tree reconstruction by applying the neighbour-joining method with Kimura’s two-parameter distance model and 1000 bootstrap replicates [11].

### Serological testing

The human serum sample was screened for MERS-CoV antibodies using a recombinant MERS-CoV spike protein subunit 1-based enzyme-linked immunosorbent assay (ELISA) kit (rELISA) following the manufacturer’s instructions (Lübeck, Germany). Camel sera were screened using MERS-CoV S1 IgG-specific antibodies semi-quantitative ELISA kit according to the manufacturer’s recommendations (EUROIMMUN, Germany).

## Results

rRT-PCR results from nasal swab samples collected from the 27-year-old male rancher confirmed positive for both MERS-CoV UpE (Ct-value 31) and ORF1b genes Ct-value 30. Additionally, five samples obtained from camels in the same area also tested positive for both UpE (Ct-value 32-34) and ORF1b genes (Ct-value 28-30) by RT-PCR. However, the ELISA test yielded a negative result for MERS-CoV for the human sample and positive for all five camels involved in the study.

**Figure 1.**
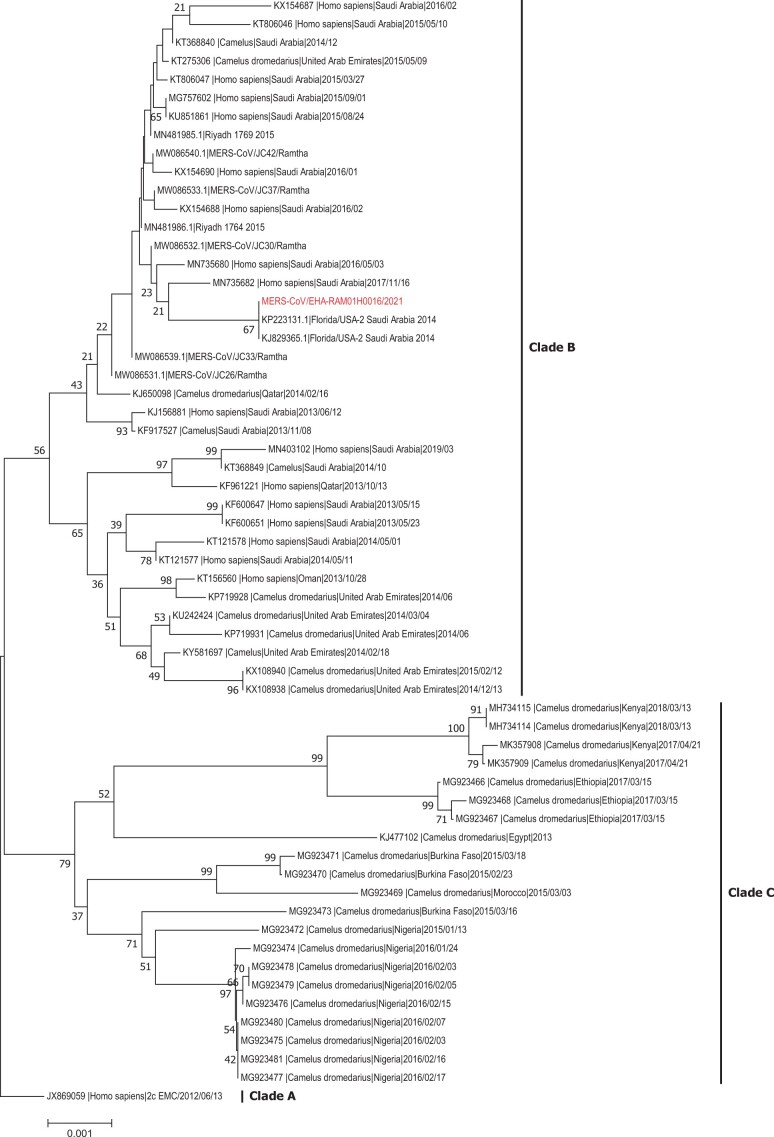
Phylogenetic analysis of MERS-CoV isolate from an asymptomatic man indicates close genetic similarity to clade B of MERS-CoV. The analysis showed that the isolate is clustered with other strains originating in the Arabian Peninsula.

The BLAST analysis of partial regions of spike gene revealed that MERS-CoV/EHA-RAM01H0016/2021 displayed the highest similarity to Florida/USA-2_Saudi Arabia_2014 and Riyadh_1764_2015 with identities of 99.79%–100%. Phylogenetic analysis revealed that the MERS-CoV/EHA-RAM01H0016/2021 spike sequence showed close identity to clade B of MERS-CoV and clustered with other strains originating in the Arabian Peninsula (Fig. 1).

## Discussion

This is the first report of a PCR-positive MERS-CoV case in an asymptomatic individual in Jordan. In 2012, MERS-CoV infection has been documented previously in Jordan in both humans and camels. The Ministry of Health of Jordan investigated a hospital outbreak of nine patients with lower respiratory illnesses [14]. The case-fatality rate was 22%, and six subjects were healthcare workers [14]. In Saudi Arabia, MERS-CoV infection was confirmed in six patients admitted to the hospital with severe respiratory symptoms in 2014 [14]. Three of these patients with comorbidities died during the study period, while three had successful outcomes [6]. Another study confirmed MERS-CoV infection by rRT-PCR in a nasal swab from a deceased 26-year-old female with high fever and severe respiratory symptoms in the Western Province region of Saudi Arabia [1]. MERS-CoV has sporadically spread among people, with more severe disease observed in older males with underlying conditions [2]. Reports of MERS-CoV outbreaks in Jordan and elsewhere suggest that hospital spread is a significant factor, as more than one-fifth of documented cases involved healthcare workers [2].

In camels, a recent review article found that seroprevalence and viral RNA prevalence in dromedary camels were highest in West Asian countries, with estimated rates of 77.53% and 23.63%, respectively [15]. The study also indicated that oral samples had the highest prevalence of MERS-CoV RNA (45.01%) compared to rectal swabs (8.42%). Females had higher seroprevalence and viral RNA prevalence, while local camels had lower rates [15]. In Jordan, a comprehensive longitudinal surveillance study conducted in two phases revealed the widespread presence of MERS-CoV in camels [16].

This case report is a direct outcome of a comprehensive multidisciplinary One Health approach that integrated a large cohort of human and animal health data within a longitudinal framework to evaluate the dynamics of MERS-CoV transmission in Jordan from 2019 to 2025. Although specific contact details of the man infected with MERS-CoV in this case report are lacking, the genetic analysis of the virus added a depth to the findings by revealing the potential sources and evolution of the strain being under study in this case report. The connection between human MERS-CoV and dromedary camels was established in 2014 [14–18]. MERS-CoV is considered endemic in camels in the Arabian Peninsula, where camels are not only used for livestock but also for racing and beauty contests, giving them high value [19]. Consequently, the management systems for such camels in this region differ from those elsewhere [20]. The camel density and movement patterns are also unique in this region, with little control over human or animal movement across the farms [21]. In most of these regions, camels are kept in high densities without biosecurity measures and without quarantine for incoming camels during the racing season [5]. Similar management practices and camel movements are observed in Jordan. The local camel owners’ knowledge, attitudes, and practices may contribute to the high prevalence of MERS-CoV in the Middle East, as they often ignore control recommendations due to a perceived low risk [22].

## Conclusions

This case report documents the first PCR-positive detection of MERS-CoV in an asymptomatic individual in Jordan, suggesting that the virus is actively circulating within the population.

## Data Availability

The data underlying this article will be shared on reasonable request to the corresponding author. Key points MERS-CoV was detected in an asymptomatic man in Jordan in January 2021 using RT-PCR. Phylogenetic analysis placed virus in clade B, linked to Arabian Peninsula strains. Camels have been implicated as potential source, highlighting zoonotic transmission significance. Continuous surveillance crucial for early detection and containment of MERS-CoV outbreaks.
